# Management of Small Renal Masses: Literature and Guidelines Review

**DOI:** 10.1590/S1677-5538.IBJU.2025.0203

**Published:** 2025-05-10

**Authors:** Antonio Silvestri, Filippo Gavi, Maria Chiara Sighinolfi, Simone Assumma, Enrico Panio, Daniele Fettucciari, Giuseppe Pallotta, Or Schubert, Cristina Carerj, Mauro Ragonese, Pierluigi Russo, Riccardo Bientinesi, Nazario Foschi, Chiara Ciccarese, Roberto Iacovelli, Bernardo Rocco

**Affiliations:** 1 Fondazione Policlinico Universitario Agostino Gemelli Department of Urology Rome Italy Department of Urology, Fondazione Policlinico Universitario Agostino Gemelli, IRCCS, Rome, Italy; 2 Fondazione Policlinico Universitario Agostino Gemelli Department of Oncology Rome Italy Department of Oncology, Fondazione Policlinico Universitario Agostino Gemelli, IRCCS, Rome, Italy

**Keywords:** Kidney Neoplasms, Watchful Waiting, Adenocarcinoma, Clear Cell

## Abstract

Renal cell carcinoma (RCC) ranks among the most prevalent malignancies worldwide, with a rising incidence attributed largely to the incidental detection of small renal masses (SRMs ≤ 4 cm) through widespread abdominal imaging. Historically managed with radical nephrectomy, treatment of SRMs has evolved significantly over recent decades. Partial nephrectomy has become the standard surgical approach, while active surveillance (AS) has emerged as a viable alternative for select patients, particularly those with comorbidities or limited life expectancy. AS involves serial imaging to monitor tumor progression, reserving intervention for signs of clinical advancement.

This review synthesizes oncological outcomes and current management strategies for SRMs, comparing AS with immediate intervention. A comprehensive literature search (2005–2024) was performed across PubMed, Web of Science, and Scopus, complemented by an analysis of major international guidelines (EAU, AUA, ESMO, CUA, and Latin American Renal Cancer Group). All guidelines support AS for selected patients with cT1a tumors, though criteria vary. The AUA limits AS to tumors <2 cm, while only its guidelines define clear triggers for transitioning from AS to treatment. Imaging surveillance intervals and biopsy indications also differ, with broader support for renal mass biopsy prior to ablation but more selective use during AS.

This review underscores the importance of individualized decision-making in SRM management and highlights areas of consensus and divergence among contemporary guidelines.

## INTRODUCTION

According to the Global Cancer Observatory, renal cell carcinoma is the 14th most common malignant tumor, precisely the 9^th^ most common cancer among men and the 14th among women, with 431,288 cases in 2020 (1). RCC incidence is higher in Europe and North America and has been increasing in the last decades. Simultaneously the mortality rate in developed countries has declined. It has been hypothesized that this phenomenon is related to the widespread use of abdominal imaging for nonspecific musculoskeletal or gastrointestinal complaints, leading to the incidental detection of otherwise asymptomatic small renal masses (≤ 4 cm) (2, 3). Traditionally, RCC was treated with radical nephrectomy (RN), regardless of renal mass dimensions. However, the management of small renal masses (SRMs) has undergone a significant transformation over the past decades. Nephron sparing surgical approach, such as partial nephrectomy (PN), has become the standard treatment, and active surveillance (AS) has moved from being a niche approach to an established treatment option for a specific patient population. AS is defined as the initial monitoring of tumor size by serial abdominal imaging (US, CT, or MRI) with delayed intervention reserved for tumors showing clinical progression during follow-up (4). This review aims to resume oncological results on the management of small SRMs with either AS or immediate treatment, focusing on the key factors influencing the choice between these two strategies.

## MATERIALS AND METHODS

A comprehensive literature review was conducted to identify studies published in English between 2005 and 2024 focused on the management of small renal masses (SRMs). PubMed, Web of Science, and Scopus databases were queried using the following key words: "small renal mass", "active surveillance", "treatment" and "renal mass biopsy". A review of international available guidelines was performed as well, to depict the definition of SRM, guidelines’ position on active surveillance, definition of active surveillance monitoring.

## SUMMARY OF THE CURRENT GUIDELINES

At present, EAU, ESMO and CUA consider active surveillance in cT1a RCC, while AUA recommends active surveillance only for SRM < 2 cm. EAU, AUA, ESMO, CUA and Latin American Renal Cancer group agree on the patient selection, suggesting active surveillance to frail and comorbid patients, with the rationale that primary intervention would overweigh oncological benefits. AUA, CUA and Latin American Renal Cancer group suggest repeated imaging every 3-6 months during the first year, then every 6-12 months. Instead, EAU and ESMO do not specify any imaging protocol for active surveillance. Only the AUA guidelines provides specific triggers for a change in the disease management from AS towards intervention, which are tumor size > 3 cm, stage progression, growth kinetics > 5 mm/year, clinical changes in patient/tumor factors, additional biopsy results. Recommendations for renal mass biopsy (RMB) vary among the guidelines. EAU, AUA and CUA agree on practicing RMB before ablation treatment in SRM. RMB is recommended in AS, according to EAU and ESMO, only for selected patients, and according to AUA, only in the suspicion of non-malignant lesions. An overview of the summary of the current guidelines is presented in Table-1.

**Table 1:** Overview of the summary of the current guidelines.

	Inclusion criteria	Patient selection	Imaging	Triggers for intervention	Renal mass biopsy
**EAU**	cT1a RCC	Frail and comorbid patients	No imaging protocol	/	Before ablation treatment
**AUA**	SRM < 2cm	Frail and comorbid patients	Every 3-6 months during the first year, then every 6-12 months	Tumor size > 3 cm, stage progression, growth kinetics > 5 mm/year, clinical changes in patient/tumor factors, additional biopsy results	Before ablation treatment and in suspect of non malignant lesions
**ESMO**	cT1a RCC	Frail and comorbid patients	No imaging protocol	/	Only in selected patients
**CUA**	cT1a RCC	Frail and comorbid patients	Every 3-6 months during the first year, then every 6-12 months	/	Before ablation treatment
**Latin American Renal Cancer Group**	Small tumors	Frail and comorbid patients	Every 3-6 months during the first year, then every 6-12 months	/	/

## EVOLUTION OF THE USE OF ACTIVE SURVEILLANCE

Initially, SRMs were almost exclusively managed with an interventional approach, which included RN or PN. However, as the incidental diagnosis of SRM increased through advanced imaging techniques such as ultrasound, computed tomography (CT), and magnetic resonance imaging (MRI), it became apparent that many of these masses had indolent or even benign behavior. This awareness led to a reconsideration of the management of SRM and the gradual introduction of AS as an alternative option. In the 2000s, early retrospective studies began to examine the natural history of SRM and the outcomes of conservative management. These studies showed that many SRM grow slowly and have a low risk of metastasis, paving the way for AS as a viable option (5–7). Since the 2010s, several prospective studies have further consolidated the role of AS. The Delayed Intervention and Surveillance for Small Renal Masses (DISSRM) registry, a multicenter prospective study, has shown that AS is non-inferior to primary intervention in terms of cancer-specific survival (CSS) at an intermediate follow-up of 5 years (8). Initially AS was considered primarily for elderly patients with significant comorbidities, in whom the risks associated with surgery may outweigh the benefits (9–11). Few years later Metcalf et al. focused the attention on AS in young and healthy patients, showing that even in patients aged less than 60 years AS in SRMs is not inferior to immediate intervention in terms of overall and cancer-specific survival. Nowadays the management of SRMs through AS is widely recognized by international guidelines.

## ACTIVE SURVEILLANCE VS. IMMEDIATE TREATMENT

The rationale for managing RCC with AS derives from the observation that up to 20-30% of RM < 4 cm are histologically benign, and those that are malignant often exhibit a low degree of aggressiveness (12, 13), characterized by a slow growth rate and a low metastatic potential, with a progression to metastatic disease observed in only 1-2% of cases (14). Initial tumor size at diagnosis does not reliably predict the natural history of renal masses, although malignant lesions may exhibit a higher growth rate (14). Kouba et al. demonstrated that, among SRMs managed with AS, those who underwent delayed intervention exhibited a higher tumor growth rate (6). Delayed intervention does not result in an increased risk of disease progression, is not associated with added surgical morbidity, and does not preclude patients from undergoing definitive surgery via a minimally invasive approach with comparable oncological outcomes (5, 7). Therefore, a deferred intervention is a safe approach in the management of SRMs (15, 16). Delayed intervention may include PN, RN or image-guided percutaneous ablation, such as cryoablation (PCA), radiofrequency ablation (RFA) or microwave ablation (MWA) (17, 18). Five studies concur on establishing a growth rate > 0.5 cm/year as a threshold for delayed intervention (8, 11, 19–21), while three of these also consider a tumor diameter > 4 cm as an additional criterion for intervention (8, 19, 21).

The Delayed Intervention and Surveillance for Small Renal Masses (DISSRM) registry is a prospective study designed for patients with SRMs undergoing either AS or primary intervention. The DISSRM protocol advises serial imaging every six months for two years, followed by annual imaging thereafter. Axial imaging, utilizing either computed tomography (CT) or magnetic resonance imaging (MRI), is performed within six months of enrollment in the registry. Contrast-enhanced imaging is employed in patients with adequate renal function (11). Ultrasound may be considered every twelve months for a duration of three years. The use of CT or MRI remains at the discretion of the physician in cases of ambiguous ultrasound findings or observed changes (8). Similar protocols have been adopted by other investigators; for example, Marchioni et al. implemented abdominal imaging every four to six months for two years, succeeded by imaging every six to twelve months (9). An overview of the included studies to evaluate the efficacy of AS in SRMs is presented in Table-2.

**Table 2:** Characteristics of the studies included in the review to evaluate the efficacy of AS in SRMs.

First Author (Year)	Study Type	N (Patients with RM)	N (AS Group)	Mean Age	Inclusion Criteria	Follow-up (Years)	N of patients who underwent renal mass biopsy	N of AS patients who underwent delayed intervention	Growth Rate (cm/year)	Development of Metastatic Disease	5-Year OS (%) in AS	5-Year CSS (%) in AS	Key Findings
Kouba, et al. (2007) (6)	Retrospective	545	43	67	Diagnosis of RM	3	/	13	1.01	/	/	/	Watchful waiting for RMs is a valid option for selected patients, and an eventual delayed intervention does not have a negative impact on pathological outcomes
Crispen, et al. (2008) et al. (7)	Retrospective	82	82	64	RM ≤ 4 cm	1.8	25	82	0.30	No	/	/	Small renal tumors have a slow interval growth, and their management can be delayed without limiting available treatment options or incurring high disease progression
Rais-Bahrami, et al. (2009) (5)	Retrospective	32	32	59	Incidentally discovered RMs who underwent laparoscopic partial nephrectomy	1.3	/	32	0.56	/	/	/	A delay in surgery of SRMs of > 1 year does not preclude patients from undergoing definitive surgery via a minimally invasive approach with an equal oncolgical outcome
Zini, et al. (2009) (29)	Retrospective	10 292	433	61.9	RCC ≤ 4 cm treated with either nephrectomy or non-surgical management	4.2	/	/	/	/	/	/	Non-surgical management of RMs has higher RCC specific mortality rates than nephrectomy
Lane, et al. (2010) (27)	Retrospective	537	105	75	cT1 renal tumors	3.9	/	/	/	Yes (n=26)	76	/	In patients > 75 years, surgical management of clinically localised renal cortical tumors is not associated with increased survival
Brunocilla, et al. (2014) (10)	Retrospective	42	42	75	Contrast-enhancing SRMs suspicious for RCC	5.8	15	12	0.8	Yes (n=2)	/	/	Faster growth rates in SRMs could be an expression of malignant disease, suggesting delayed surgical intervention. AS is an option for the management of SRMs in low life expectancy patients
Patel, et al. (2012) (30)	Retrospective	234	71	71.9	T1a SRMs managed with AS, RN, or PN	2.8	7	14	0.21	Yes (n=1)	83	98.6	AS of SRMs offers oncological efficacy equivalent to surgery in the short/intermediate term
Brunocilla, et al. (2013) (14)	Retrospective	62	62	75	Contrast enhancing SRMs suspicious of RCC.	7.6	25	20	0.4	Yes (n=2)	/	/	Most SRMs have an indolent course, and AS is an option for selected patients
Sugimoto, et al. (2013) (24)	Retrospective	292	32	63.7	cT1aN0M0 SRMs managed by immediate or delayed intervention	2.2	/	32	/	/	72.6	87.5	Delayed surgery for SRMs is a treatment option, and has non inferior overall survival rate compared to immediate surgery
Pierorazio, et al. (2015) (8)	Prospective	497	223	70.6	cT1a SRM on axial imaging	2.1	32	21	0.11	No	75	100	AS is not inferior to primary intervention
Danzig, et al. (2015) (26)	Prospective	162	68	71.7	SRMs managed with either AS or PI, with respectively preoperative/postoperative or 2 consecutive serum creatinine values	1.5	/	/	/	/	/	/	Patients in AS have superior eGFR rate preservation than those who undergo RN, but no significant difference than those who undergo PN
Bazan, et al. (2018) (12)	Retrospective	82	82	77	Contrast-enhancing (> 20 HU) RMs ≤ 4 cm (cT1aN0M0) or renal cysts (BosniakIIF-IV)	4.6	0	5	/	No	/	/	AS is a safe option for the management of SRMs
Gupta, et al (2018) (11)	Prospective	371	371	71.3	RMs ≤ 4 cm undergoing AS or primary intervention	2	52	46	0.18	No	/	/	AS is a safe management option, but counseling is essential to determine suitability of patients
Petros, et al. (2019) (21)	Prospective	272	272	68.5	SRMs ≤ 4 cm	4.8	105	64	0.24	Yes (n=4)	73	98	Survival of patients with SRMs < 3 cm on AS improves after the initial 2 years, suggestinf role for re-counselling those who survuve the 2 year landmark
Alam, et al. (2019) (18)	Prospective	638	339	70.6	SRMs ≤ 4 cm managed with AS, ablative therapy, PN, or RN.	3.0	11	46	/	Yes (n=2)	66.1	100	AS is a reasonable option for selected patients (old patients with multiple comorbidities)
Tan, et al. (2020) (16)	Retrospective	14 677	627	55	Patients < 70 years with cT1aN0M0 RCC and Charlson Comorbidity Index 0	6.9	/	627	/	/	89.9	/	No significant difference in OS between immediate nephrectomy vs. delayed nephrectomy, suggesting that a period of observation is safe to allow identification of RMs that will benefit from surgery.
Marchioni, et al. (2021) (9)	Retrospective	483	121	80	Patients ≥ 75 years with SRMs ≤ 4 cm managed with AS or PI	2.3	/		/	/	70	/	AS is an appealing treatment for very elderly patients with SRMs, and it does not compromise survival outcomes
Jakubowicz, et al. (2022) (22)	Prospective	106	41	80.5	Patients ≥ 75 years with clinically localized RMs	3.4	7	8	/	Yes (n=2)	68.3	95.1	AS is superior to watchful waiting, and should be preferred to active intervention at the beginning of the management.
Metcalf, et al. (2021) (19)	Prospective	224	82	54.6	Patients ≤ 60 years undergoing AS or primary intervention	4.9	/	13	0.09	No	90.8	100	AS is a safe initial strategy in younger patients
Umari, et al. (2022) (23)	Prospective	134	75	69.8	Single cT1a renal tumor managed with AS or percutaneous cryoablation (PCA)	3	50	12	/	Yes (n=1)	82.4	98.2	AS and PCA provide similiar outcomes and are safe and valid management options for elderly and comorbid patients with SRM2
Su, et al. (2022) (28)	Cost-effectiveness	/	/	/	Patients with SRMs undergoing either RN, PN, TA or AS	/	/	/	/	/	/	/	AS has the lowest total cost per patient among the different management options for SRMs
Cheung, et al. (2023) (25)	Retrospective	377	205	64	Patients aged 55-75 years with SRM ≤ 4 cm	4.6	100	20	/	/	95	/	AS is safe in routine clinical practice
Bertolo, et al. (2024) (15)	Prospective	356	49	66	cT1a RMs	1.5	/	49	/	/	/	/	Deferred partial nephrectomy is a safe approach in patients with SRMs
Foret, et al. (2024) (17)	Retrospective	104	/	72.2	Patients who underwent concomitant RTB and microwave ablation of SRM	1.9	104	/	/	/	/	/	Microwave ablation has shown clinical safety and efficacy in the management of RMs
Gao, et al. (2023) (31)	Retrospective	159	50	63.4	RMs ≤ 4 cm which underwent CNBs	3.4	159	/	/	/	/	/	Employment of CNB in SRMs may reveal benign diagnosis, avoiding overtreatment for benign lesions.
Finelli, et al. (2020) (33)	Prospective	134	49	70	Patients with T1aN0M0 RM, who elected not to have immediate treatment, and underwent renal mass biopsy	5.8	134	85	0.28	Yes (n=6)	/	/	Initial follow-up of histologically characterized SRMs can inform personalized treatment for patients on AS
Mazin, et al. (2024) (32)	Retrospective	195	79	70	Patients with SRMs who underwent RTB	3.5	195	/	/	/	/	/	RTB is a safe diagnostic method that provides accurate histopathological information, reducing overtreatment of benign SRMs

AS = Active Surveillance; PI = Primary Intervention; RM = Renal Mass; SRM = Small Renal Mass; RCC = Renal Cell Carcinoma; PN = Partial Nephrectomy; RN = Radical Nephrectomy; OS = Overall Survival; CSS = Cancer-Specific Survival; CNB = Core Needle Biopsy; RTB = Renal Tumor Biopsy; eGFR = estimated Glomerular Filtration Rate; TA = Thermal Ablation.

/ = indicates data not reported or not applicable

### Survival analysis

A consensus among most authors suggests that AS provides equivalent short- and medium-term oncologic efficacy to partial or radical nephrectomy for SRMs. Within the reviewed literature, six prospective studies (8, 18, 19, 21–23) and three retrospective studies (20, 24, 25) have examined either OS, CSS, or both in patients with SRMs managed with AS. The specific inclusion criteria, as well as the values of OS and CSS, are detailed in Table-1. Based on the included studies, CSS at 5 years for patients initially managed with AS is not significantly different compared to that of patients managed with immediate treatment. However, three of these studies highlighted a lower OS at 5 years in patients managed with AS compared to those managed with immediate treatment. This difference can likely be attributed to older age and a higher burden of comorbidities in the patients selected for AS (8, 18, 22), emphasizing how older patients undergoing AS will primarily die from causes other than renal cancer (22). Regarding mortality from non-RCC causes, cardiovascular events represent the leading cause of death in patients older than 75 years. Furthermore, nephrectomy is associated with accelerated renal dysfunction, which, in turn, increases cardiovascular mortality (26). Consequently, in the elderly population, active treatment is not linked to improved OS, and cardiovascular mortality surpasses cancer-specific mortality (27). According to Metcalf et al., even in a cohort of patients aged 60 years or younger at the time of diagnosis of a SRM, AS demonstrates non-inferiority to primary intervention with regards to both OS and CSS. This finding supports the possibility to expand AS to younger and healthier patients, provided they are carefully selected and monitored (19). However, the prevalent consensus suggests that active surveillance (AS) represents a reasonable strategy for elderly patients with comorbidities, whereas immediate surgical intervention, particularly partial nephrectomy (PN), may be more suitable for younger, healthier individuals (28).

Only two authors disagree on the oncological efficacy of AS in SRMs. Zini et al. report that RCC-specific mortality rates in nonsurgical management (NSM) significantly exceeds that of nephrectomy group. It is important to note that this study was not randomized, and this may limit the comparability of the NSM and nephrectomy groups due to potential selection bias and confounding factors (29). Patel et al. instead indicated that, when comparing NSM and surgical intervention, CSS was equivalent among treatment groups for patients younger than 75 years, but significantly worse for patients aged 75 years or older undergoing NSM. This discrepancy could suggest that younger patients are more effectively selected for NSM (30).

### Role of the histological diagnosis

According to several authors, the initial management of SRMs can be guided by histological diagnosis. Surgical intervention or ablation may be favored for SRMs diagnosed as renal cell carcinoma (RCC), whereas active surveillance (AS) might be preferred for indolent or benign SRMs. Consequently, renal mass biopsy (RMB) could potentially reduce overtreatment, guiding the decision to opt for AS rather than immediate treatment (31, 32). Furthermore, significant differences exist in diameter growth rate and metastatic potential between clear cell and papillary type 1 RCC SRMs, with clear cell RCC exhibiting a faster growth rate and higher metastatic potential. This highlights the potential importance of RMB in counseling patients and personalizing SRM management (33).

### Cost analysis

An analysis by Su et al. provides valuable insights into the cost-effectiveness of different management strategies for small renal masses (SRMs). The study demonstrates that the 10-year all-cause mortality rates are similar among patients managed with PN, RN, PCA, and that AS, with the option of timely delayed intervention, appears to have the lowest total cost per patient, suggesting that this strategy offers a safe and cost-effective approach to the management of patients with SRMs (28).

### Quality of life

AS patients report worse physical quality of life (QoL) than primary intervention patients, mainly due to lower scores in the physical health component (9). However, mental health scores are similar between the groups and improve over time, regardless of management strategy. This suggests that while AS may be associated with initial concerns, the mental health-related components (which include anxiety and depression) tend to improve over time, suggesting that well-selected and counseled patients may experience improved QoL. Patient selection and counseling, including a shared decision-making process, are crucial prior to initiating an AS protocol to ensure comprehensive patient understanding of the risks and benefits associated with each management option. Notably, approximately 50% of patients who elect for delayed intervention do so due to anxiety, even in the absence of significant tumor growth (11).

## CONCLUSIONS

The management of SRMs represents an evolving field, with AS emerging as a viable and safe option for selected patients. The choice between AS and immediate treatment must be individualized, considering age, comorbidities, tumor size, growth rate, and patient preferences. AS allows avoidance of unnecessary interventions and associated risks, while maintaining a safe and close monitoring to intervene promptly in case of progression. Immediate treatment remains the preferred option for young, healthy patients with fast-growing tumors or those with suspected malignancy. Renal biopsy can play a crucial role in guiding decision making by providing histologic information that can help distinguish between benign and malignant lesions and predict tumor behavior. However, it is critical to carefully consider the risks and benefits of biopsy as well as its diagnostic accuracy.

Further research, including prospective randomized controlled trials, is needed to better define the selection criteria for AS and to evaluate the long-term outcomes of different management strategies. Ultimately, the goal is to provide each patient with the most appropriate management, balancing the risks and benefits of AS versus immediate treatment, with the goal of maximizing both survival and quality of life.
